# Corporate Purpose and Democratic Theory: A Governance Trilemma

**DOI:** 10.1007/s10551-025-06127-1

**Published:** 2025-09-12

**Authors:** Rutger Claassen

**Affiliations:** Department of Philosophy & Religious Studies, Utrecht University, Janskerkhof 13, 3512 BL Utrecht, The Netherlands

**Keywords:** Corporate purpose, Stakeholders, Democratic theory, Agency theory

## Abstract

Over the last years, both in the popular press, policy and business circles and in academia, people call for corporations to orient their behavior towards ‘purpose’. This is meant as a move away from shareholder value maximization as the lodestar for corporate action. But purpose-advocates are torn between two directions in thinking about corporate governance: towards corporate governance on behalf of stakeholders by an *independent* board, and towards corporate governance by stakeholders through a *responsive* board. The paper’s aim is to enlighten this choice by placing it in a trilemma with a third option: corporate governance on behalf of shareholders. This corporate governance trilemma shows us which trade-offs are at stake in making choices between the relevant values: the minimization of externalities, collective decision-making costs and agency costs. It discusses the various trade-offs in the trilemma. Finally, the paper argues that corporate purpose is best served by a balance between board independence and responsiveness.

## Introduction

Many actors have called for corporations to orient their behavior towards ‘purpose’. This is meant as a move away from shareholder value maximization, as the lodestar for corporate action. In business circles, the American Business Roundtable (BRT), in 2019 issued a new corporate purpose statement, in which it stated that businesses should serve a broad range of stakeholders, including employees, consumers and communities. This marked an important break with the past, at least at the level of rhetoric (Harrison et al., 2020).^1^

Several academic authors have contributed to what I will call here ‘the purpose-paradigm’. In the UK, Colin Mayer and his collaborators have run a high-profile ‘Future of the Corporation’ project which has turned out two reports on purposeful business (British Academy 2020; 2019). In France, Blanche Segrestin and collaborators work on similar issues, which have inspired a change of law (the ‘loi Pacte’), introducing the possibility for companies to incorporate as a *société à mission* (Segrestin et al., 2020). Leo Strine, Elizabeth Pollmann, Rebecca Henderson and others in the US have written about corporate purpose (Henderson, 2020; Pollman, 2021; Strine, 2017). While not pretending that they agree on every issue, there seems to be a common set of commitments sufficiently clear to talk about a ‘purpose paradigm.’ This paradigm obviously has many affinities with stakeholder theories that have been proposed over the last decades (R. E. Freeman, 1984; E. Freeman & Evan, 1990; R. E. Freeman et al., 2010; Mansell, 2013). For, as we will see, at the heart of the purpose-paradigm, there is a question about how to relate to stakeholders in corporate governance.

In this paper, I want to scrutinize this paradigm from the perspective of democratic theory, a subfield in political theory/philosophy. In doing so, I conceive of this paper as an exercise in ‘business ethics as political philosophy’ (Heath et al., 2010). Democratic theory focuses on the nature, quality, forms and justifications of democratic institutions and practices, usually in the recognizably ‘political’ context of citizens in their relation to states, governments, political parties, politicians, etc. Democratic theory can serve as a source of inspiration in the economic context as well, since the concepts, arguments and theories developed in the context of the governance relation between citizens and a state can also – if with caution – be made of use in the context of the governance relation between boards and members of a corporation (Anderson, 2017; Blanc, 2023; Ciepley, 2013; Claassen, 2023; Ferreras, 2017; Frega, Herzog, & Neuhäuser, 2019; González-Ricoy, 2022; Singer, 2018). In particular, democratic theory can help us to diagnose the fact that purpose-advocates are torn between two directions in thinking about corporate governance. The most dominant interpretation is to structure purpose-driven corporate governance as a task of *independent* boards acting on behalf of a wider set of stakeholders. A vocal minority, however, argues that purpose-driven corporate governance requires democratization: stakeholders should be empowered, and boards become directly *responsive* to their stakeholders.^2^ As I will show, a similar debate can be found in the context of politics, studied by democratic theorists. If the purpose-paradigm is to become the antidote to shareholder-driven capitalism that it wants to be, this problem needs to be addressed.

My primary aim with the paper is analytical: to enlighten the choice between independent and stakeholder-responsive boards, by placing it in a trilemma with a third option: a shareholder-responsive board. This is the governance option at the heart of the Shareholder Value Maximization (SVM) paradigm, which purpose-advocates aim to replace. The resulting corporate governance trilemma shows us which trade-offs are at stake in making choices between the relevant values. The set up of the paper is as follows. In Sect. "What is the Purpose Paradigm?", I summarize the main tenets of the purpose-paradigm, and highlight its basic dilemma; whether to opt for independence or for responsiveness. In the remainder, I will scrutinize the paradigm in three steps. Sect. "Two Dimensions of Corporate Governance" introduces and discusses two dimensions of corporate governance: the constituency dimension (who are the corporation’s members?) and the control dimension (what is the distribution of power between members and boards?). Sect. "The Corporate Governance Trilemma" is the heart of the paper. It relates these dimensions through the construction of a trilemma, making use of three values: the minimization of externalities, principal costs and agency costs. The three positions emerge from combinations of these values. At this point, I provide various illustrations of how the analytical contribution of the trilemma allows us to criticize various ways in which the relevant trade-offs have been discussed in the corporate governance literature.

Finally, Sect. "The Independence-Responsiveness Trade-off" moves beyond the paper’s analytical contribution to address the normative question which option in the trilemma advocates of the purpose-paradigm should choose. It argues they should opt for a combination of responsiveness and independence, since neither in its pure form is up to the task of interpreting a business’ purpose.

## What is the Purpose Paradigm?

In this section I sketch the main contours of the purpose-paradigm, ending with the basic dilemma announced above, between independence and responsiveness in corporate governance. There are various dimensions within the discussion around ‘purpose’, some focusing more on legal interpretation, others on finance or management.^3^ My focus here will be around the normative questions whether corporations *should* incorporate a purpose, and what consequences this *should* have for corporate governance. Along these lines, I define the purpose-paradigm as consisting of three main commitments: around the purpose of the corporation, the role of the board, and the conception of control. On each of these, there is a marked departure from the SVM-paradigm.

First, corporations should be oriented towards the realization of an explicitly declared purpose. In itself, SVM could be a purpose, so to avoid making this a statement which remains indistinguishable from SVM, a sharper definition is needed. Mayer recently defined purpose as follows:

‘purpose is therefore about finding ways of solving problems profitably where profits are defined net of the costs of avoiding and remedying problems. By defining purpose and profit in this way, purpose is associated with enhancing the wellbeing and prosperity of shareholders, society and the natural world. It does not disadvantage any party because profits are only legitimate if they are not earned at the expense of other parties and corporate purposes are only valid if they are profitable in this sense.’ (Mayer 2021, 7).

The British Academy report likewise talks about a double purpose: ‘corporate purposes should profitably solve problems for people and planet, and avoid profiting from creating problems for people and planet.’ (British Academy 2020, 10). The concept of a ‘problem’ is prominent in this statement, but not particularly helpful. Adherents of SVM argue that profit-oriented firms fulfill consumer needs and by doing so provide wages to employees, business opportunities to suppliers, and dividends to shareholders. They solve lots of problems. As a shortcut, I will refer to the purposes relevant to the purpose-paradigm, as those purposes which are not already realized by SVM-driven business practices; i.e., as *externalities*.^4^ The first part of the statement then refers to positive externalities, the second part to negative externalities.

For a business, drawing up a purpose is aspirational, and requires implementation in the business strategy. The extent to which negative and/or positive externalities are internalized in the actual business strategy, can vary, from more radical to more cautious approaches. One can hence construe a continuum of various relations between profits and social value as the objective of the firm (Lankoski & Smith, 2018). Mayer’s statement of purpose is broad and accommodates various points along this continuum. For convenience’s sake, in the construal of the trilemma (Sect. "The Corporate Governance Trilemma"), I will refer to ‘minimizing negative externalities’ as the relevant purpose. One can however extend this to include the promotion of positive externalities as well. Moreover, one can imagine this purpose to have various strengths, in terms of the trade-off with profitability, along the continuum.

Second, directors’ duties should be oriented towards the purpose of their corporation. SVM, which rests on agency theory’s conception of directors as agents for the wishes of their principal, the shareholders (Jensen & Meckling, 1976) posits a fiduciary relation between directors and shareholders, while the purpose-paradigm constructs a fiduciary relation between directors and the corporation itself. Not a concrete set of persons (shareholders) but an abstract purpose, becomes the object of the fiduciary relationship. Sometimes, it is said that the fiduciary duty is ‘to the corporation’. Now this is a separate legal person, which can only come to act in social and economic life by being represented in the actions of natural persons. The directors *are* the representatives of the corporation. The purpose-paradigm proposes to understand the abstract purpose as the animating heart of the corporation, and the directors’ fiduciary duty as being to realize this purpose.

Third, what about control of the board? Here purpose-advocates agree in their critique of the SVM-paradigm, which advocates exclusive control by shareholders. But what should take its place? It is here that we see the dilemma between two ‘wings’ of the purpose-paradigm appear.^5^ Kevin Levellain and Blanche Segrestin have argued, that ‘profit-with-purpose corporations’ (PPC’s) represent a new paradigm, which cuts a third way compared to the familiar shareholder and stakeholder theories of the corporation. These antagonists, they suggest, are both beholden to a ‘primacy’ or a ‘political’ way of looking at the corporation, that is ‘fundamentally focused on the appropriate distribution of rights between constituencies’ so that ‘it leads to boiling corporate governance down to the questions of “who elects whom?” and “who monitors whom?”’ (Levillain & Segrestin, 2019, 642). By contrast, the PPC paradigm they describe as follows:

‘The existence of a common purpose, explicitly stated in publicly available legal documents, enables derivation of objective and stable criteria for controlling executives' action – for instance through the definition of common standards – *independently from the party that is supposed to exert this control*. The pursuit of a responsible purpose no longer depends on the contingent engagement of one particular leader or the consent of changing shareholders: it is made *independent* from the specific groups of constituencies given “political” rights in the company's governance system.’ (Levillain & Segrestin, 2019, 642)[italics added, R.C.].

Although the orientation to common purpose doesn’t do away with the need to define control rights, nonetheless, the purpose-corporation provides a profoundly different way of thinking about corporate governance. Not the relation between different groups, but their common relation to an abstract purpose is at the heart of the corporation.

However, this portrayal is by no means shared by everyone. Others argue that control rights need to be assigned to a broader range of stakeholders, hence calling for a democratization of the corporation (Aytac, 2025). For example, Gerald Davis argues that the purpose movement needs to embrace the empowerment of employees: ‘Let’s give democratic control to those who do the real work’ (Davis, 2021, 909). Leo Strine explicitly laments the absence of ‘power’ in the analysis of purpose:

‘Corporate directors cannot subordinate the best interests of stockholders to those of other corporate constituencies unless the stockholders themselves support that subordination. And when a conflict emerges between the interests of corporate constituencies without power within the corporate polity—which is all of them other than stockholders—and the one with power—the stockholders—those elected by the stockholders bend to the will of their citizenry. And their citizenry is by statute those who hold the corporation’s voting equity.’ (Strine, 2017, 179).^6^

On these and other analyses, the political dimension of corporate governance cannot be transcended by a commitment to an abstract purpose. Purpose must be aligned with stakeholder governance after all; or alternatively, it must be put into the hands of shareholders, who are to act as the trustees of a wider set of stakeholders (Mayer, 2020). We are back at the primacy approach which characterized the shareholder versus stakeholder debate.

In this paper, I want to shed light on this dilemma. My method will be to turn it into a trilemma. But to construct this trilemma, we first need to disentangle two basic dimensions of corporate governance, that are not always as neatly separated as they should be.

## Two Dimensions of Corporate Governance

In this section I introduce two dimensions of corporate governance: the constituency dimension, and the control dimension. Both dimensions have been discussed in the corporate governance literature in law and economics. But we can get a better handle on these dimensions by starting elsewhere, in political theory.

In political theory, the constituency and control dimensions are debated in the context of the state. We can capitalize on these discussions, since the state, like the corporation, is an abstract legal person for organizing collective action of groups of members/citizens. Indeed, the history of corporation theory from the Middle Ages teaches that there have been multiple points at which the development of the corporation (*universitas* in Roman law) was a source of inspiration for thinking about states as well (Ciepley, 2017; Tierney, 1982). In both contexts, there are two main governance dimensions. The constituency dimension relates to the question who is allowed to be a member (or citizen) of the corporation (or state), i.e., it is about the identity of the members. This is the horizontal dimension of corporate governance: determining the member base. The control dimension relates to the question as to the distribution of power between the member base and the central governing body governing over the members (the board of directors in a corporation, the government in a state). This is the vertical dimension of corporate governance.

In the constituency dimension, the membership of a corporation can include fewer or more groups with a different identity. We can think of this dimension as a spectrum running from a small constituency basis to a larger one. A smaller, more exclusive membership basis makes the corporation more *homogeneous*. A larger, more inclusive basis makes the corporation more *heterogenous*. In political theory this dimension has been the subject of debate throughout the centuries, with respect to the issue of voting rights in politics. Originally, only white, property-owning males could vote. Later this was broadened to all males and finally to women as well. Debates today are about voting rights for children, animals, future generations, and immigrants. These questions of inclusion are discussed in political theory under the heading of the ‘boundary problem.’ (Miller, 2020). The problem is that one cannot legitimately determine the boundaries of the political community (hence state) on the basis of a decision by its *current* members, since who are to be its members is what is at stake in the first place. Various principles have been proposed to solve this quandary. One is the so-called ‘all-affected’ principle: only all those affected by the decisions of the state/community should be included in its decision-making processes.

In the corporate context, we detect a similar boundary problem (González-Ricoy & Magaña, 2024; Stehr, 2022). The question of membership can also be transposed in the terminology of agency theory: which parties should be recognized as the principals of the corporation?^7^ Agency theorists have argued that only shareholders are to be seen as such. Shareholders, amongst all other stakeholders, have a special position (Fama & Jensen, 1983; Jensen & Meckling, 1976). This position is confirmed in the corporate law of those jurisdictions in which shareholders have the exclusive right to elect and control the board. In the construction of agency theory, this allocation of voting rights expresses and supports the basic normative position: boards are to act as agents towards their shareholders as their principals.^8^ However, agency theory does *not* imply shareholder primacy (Heath, 2009, 506). The principal-agency framework can also be used in combination with models of the corporation in which the constituency basis is broadened beyond shareholders (Lan & Heracleous, 2010; Sjåfell, 2018). With increased inclusivity comes enhanced heterogeneity of interests. The choice of a broader multi-principal constituency basis also has repercussions on the second dimension of corporate governance, i.e., the control dimension.

On the control dimension, the central issue is how to organize the relation of representation between a board and the members (Claassen, 2024). In political theory, this has been discussed under the heading of the ‘mandate-independence controversy.’ Hannah Pitkin, in her classical study of representation, distinguished two ideal–typical positions, which we can see as the extremes of the control spectrum (Pitkin, 1967). On the one hand boards/governments (or parliamentarians, in Pitkin’s rendering) can be seen as trustees of their electorate. If so, they operate *independently* from their electorate, judging themselves what is in the interests of their voters.^9^ On the other hand, boards (or parliamentarians) can be seen as obeying the expressed preferences of their electorate, which reach them at every step as clear instructions. Here they are operating as delegates, or *responsive* agents. The spectrum, then, is one along which the intensity of the control exercised by the principals to align their interests with that of their agents increases.^10^ One can either give more control rights to principals or leave control to agents, or anywhere in between – the structure of the allocation of control rights over these two parties is zero-sum, and constitutes the independence-responsiveness spectrum.^11^

From the tradition of corporation theory, we have examples of both extremes of the spectrum. As David Ciepley explains, the Roman law corporation is the exemplar of a democratic corporation, with a majority of members holding decision-making authority, to which the corporate head remained accountable. The Catholic Church in the Middle Ages would invent the canon law corporation, a more autocratic model of corporate governance. In such a corporation, the head (i.e., a bishop) would share decision-making power with the members (i.e., cathedral canons). The head’s election for life would diminish his accountability to the members (Ciepley, 2017, 421).

In today’s context, agency theory understands the relation between shareholders and boards as potentially conflictual. Due to the separation of ownership and control, boards of large companies operate at considerable distance from their shareholders. The latter have insufficient time, information and capacities to control whether boards act in their interests, or whether they abuse their position to act in their own interest. Hence, agency theory prescribes measures to effectively control boards, so that they act in the interests of shareholders. On the control dimension, agency theory therefore fears the independent pole of the spectrum, where CEO’s use their power for self-aggrandizing projects and/or shirking their jobs. To counteract these threats, agency theory opts for a position close to the responsive pole. Control is effectuated by powerful (institutional) activist shareholders, who pressure boards to adopt the policies they favor, use the threat of firing boards who don’t listen to their demands, adopt executive remuneration schemes aligning the personal interest of directors with shareholder value, etc.^12^

With these two dimensions at hand, we can analyze the dispute between the SVM-paradigm and the purpose-paradigm as follows. The SVM-paradigm puts forward a small, homogenous constituency basis (shareholders only) and combines this with a preference for responsive principal-agent relations. The purpose-paradigm aims to broaden the constituency basis, via an enlarged purpose which benefits others than shareholders as well. In this paper I do not take a stance on how much this constituency basis should be broadened (and whether firms should be free to decide this for themselves, or whether law should nudge or direct them to a certain position on the constituency dimension). I remain agnostic on whether the all-affected principle or some other principle(s) should be used to determine this, and on how many or which stakeholder groups should be included as a result of the application of such a principle. The focus in this paper is on the consequences of broadening of the constituency basis (whether to a greater or to a lesser extent), for the control dimension. A move on the constituency dimension does not pre-determine a position on the control dimension. Should these new principal groups get effective control (responsiveness to them) or are they to be kept at some distance from the board, so that it can freely implement its interpretation of the abstract purpose (independence)? The two wings of the purpose-paradigm part ways on that question, as we saw earlier.

This debate is not merely an abstract, academic one. In various countries, statutes have been adopted to create a new legal form for social enterprises, that want to combine profits with a purpose-orientation. These go under different names, *Benefit Corporations* in the US, *Community Interest Companies* in the UK, *sociétés à mission* in France. In each of these statutes, design choices have been made between independence and responsiveness of boards. For example, US Benefit Corporations need to adopt a third-party standard developed by an independent entity, to verify whether and to what extent boards have realized the purpose of the corporation. The company can choose its own standard and the company’s report assessing its performance under the standard does not need to be verified by a third party auditor (Clark Jr. & Babson, 2012, 38:842–48). This set of arrangements is very different from the UK approach. The performance of a Community Interest Company on its purpose is supervised by a public body, the Regulator of Community Interest Companies, who must evaluate the annual report of the company in light of the ‘community interest test’. It however so far adopts a ‘light touch’ approach and rarely makes public statements about company’s performance (Boeger et al., 2018, 352). In France, yet a different set of arrangements prevails, for the law specifies that *sociétés à mission* must create a special committee within the company, which monitors and evaluates the performance of the company on its mission (Segrestin et al., 2020, 8).

Yet another example of purpose-governance is given by companies owned by industrial foundations or trusts. These companies – in contrast to the various social enterprise models discussed above – also adapt their ownership structure to their purpose-orientation. Foundation-owned companies have existed for a long time in Denmark, and to a lesser extent in Germany (Thomsen & Kavadis, 2022; Sanders & Thomsen, 2023). Shares with voting rights are assigned to a non-profit foundation. The foundation board is charged with monitoring the company board on its realization of the company’s purpose. Shares with profit rights are either given to the same or to another non-profit foundation.^13^ In this way, profits do not have to be paid out to satisfy the commercial interests of outside investors but can be reinvested in the company (or spent as charity by the foundation). The purpose of the company takes pre-eminence. Recently, the phenomenon has also become known as ‘steward-ownership’ of companies (Purpose Foundation, 2017; Sanders, 2022).^14^ Patagonia’s 2022 conversion to steward-ownership brought the phenomenon to the attention of a US audience.^15^ Another example is OpenAI, whose corporate governance problems I will use as an illustration below (see Sect. "The Independence-Responsiveness Trade-off").

This all-too-brief overview illustrates that purpose-oriented companies, and the laws designed to facilitate their adoption, are currently experimenting with different governance arrangements. Some of them exemplify board independence, others bring in stakeholder responsiveness to varying degrees. Hence the choice under discussion is definitely not a merely academic one. This brief overview already shows a bewildering set of options to control the purpose-orientation: by a public body (UK), by an internal committee (France), through a self-assessment based on a third-party standard (US) or by a separate foundation. A more elaborate institutional analysis (which I cannot make here) would have to position each of these arrangements on the responsiveness-independence spectrum, by studying the extent to which board decision-making is constrained and controlled by these various ‘purpose-controlling bodies’ (my term).

Moreover, the overview also shows that there is an additional complexity in the case of purpose-governance. As we saw, purpose-governance often introduces a new purpose-controlling body, either external to or embedded in the company. This implies the creation of a *double* relation: between the purpose-controlling body (any of the just-mentioned options, except for the case of self-assessment) and the board of the company, and between the stakeholders (mentioned in, or implied by the purpose) and the purpose-controlling body. *Both* relations can lean either towards independence or to responsiveness. For maximal responsiveness to stakeholders, stakeholders need to be able to effectively control the purpose-controlling body *and* the latter needs to be able to effectively control the board of the company. For example, in the case of foundation ownership, there is the relation between the foundation board on the one hand and the company board on the other hand. This relation can be governed in the same way as the relation between holders of voting shares and the company board in a regular company.^16^ The second relation is between the foundation board and the stakeholders of the company. This relation is marked by independence where foundation boards are self-appointing (cooptation). But there are also models where steward appointments need to be confirmed by worker councils, or a separate trust which governs on behalf of workers, as is the case in the John Lewis Partnership (Purpose Foundation, 2017, 18, 23). The latter arrangement makes the board (indirectly) more responsive to workers (and this model could be extended to other stakeholders).^17^

With this overview in mind, let’s now move to a systematic exposition of the trilemma which, I aim to show, arises once we bring together the constituency and control dimensions of corporate governance.

## The Corporate Governance Trilemma

**Fig. 1:**
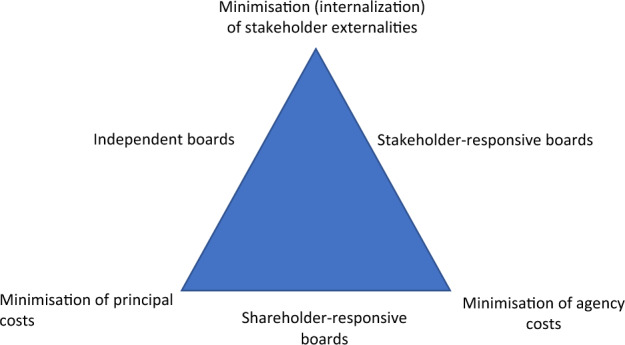
The Corporate Governance Trilemma

When we combine the two corporate governance dimensions presented in the previous section, we can systematize the problem of corporate governance as a choice between three positions (see Fig. 1).^18^ As in any trilemma, the choice situation presents us with three desirable values. Any combination of two of these values can be realized in practice, but not all three together.^19^ Hence, we face an inevitable trade-off between three values, leading to three policy options which each realize two of them. All these values and policy options are ideal types which can be approximated to a greater or lesser extent. In the following I present the ideal types. But it should be kept in mind that the triangle represents a policy space. In practice, a corporate governance system can occupy any position within the triangle by mixing the three ideal–typical policy options identified here. The fact that intermediate positions are possible, however, doesn’t mean that the trade-offs between the underlying values is canceled. Moving away from one of the horns of the triangle still means a (partial) sacrifice of the value represented by that horn.

Each of the three values can be stated in economic terms (see Sect. "The Independence-Responsiveness Trade-off"). These are principal costs, agency costs, and externalities. These will be explained as we go along. The three policy options each put another party in the position of principals of the board: corporations, shareholders or stakeholders. Boards responsive to shareholders combine low principal costs with low agency costs. Independent boards combine low externalities with low principal costs. Boards responsive to stakeholders combine low externalities with low agency costs. It may perhaps be surprising that this paper doesn’t restrict itself to the latter two options (presenting a dilemma rather than a trilemma), since an advocate of the purpose-paradigm will by definition not be interested in adopting the first option (shareholder-responsive boards). However, this regime is the currently dominant one. Taking this as the baseline, purpose advocates propose to make a more or less strong move in the direction of internalizing externalities (see Sect. "What is the Purpose Paradigm?" on the ‘continuum’). Hence the policy space of the trilemma is necessary to describe the space in which they will land, diverging from the currently dominant option of shareholder-responsiveness more to the extent that their advocacy of purpose becomes more radical.

As this overview shows, the trilemma works with a distinction between agency costs and principal costs. In Jensen and Meckling’s analysis, agency costs are the costs a principal suffers because control is delegated to an agent. The main source is the self-seeking behavior of agents (managers), which leads them to shirk or otherwise divert firm resources to themselves. As a result, the welfare of principals is lower than it would otherwise have been. The ‘direct costs of managerial self-seeking behavior’ (Goshen & Squire, 2017, 776) represent the welfare loss for principals because of a misalignment between their interests and the self-interest of their agents. To minimize this, principals can monitor their agents, i.e., engage in monitoring costs: the expenditures principals make to monitor the agent’s behavior. Agents can spend bonding costs, to convince their principals that they act in their best interests. Together, these three types of costs represent all agency costs (Jensen & Meckling, 1976, 308).

Jensen and Meckling refer to the direct costs as the ‘residual loss’, to reflect the fact that even with optimal monitoring and bonding costs, agents do not perfectly reflect the wishes of their principals. However, Goshen and Squire’s use of the term direct costs puts them upfront. Monitoring and bonding activities mitigate these costs, but have their own costs. Monitoring is a form of activity which brings control (partially) back to principals themselves. Goshen and Squire introduce the concept of ‘total control costs’, which encompasses the costs of letting the agent exercise control (‘agency costs’) *and* the costs of letting the principal exercise control (‘principal costs’). Control they define as all activities to ‘select the business strategy and then execute it’ (Goshen & Squire, 2017, 783). Control can be completely in the hands of principals – then there simply is no delegation to agents. This may be costly, for example because principals are (relatively) incompetent. Incompetence is one important source of principal costs. To decrease these costs, principals may hire (more competent) agents. This decreases principal costs, but it introduces agency costs.

Goshen and Squire argue that principal costs and agency costs need to be balanced: ‘Principal costs and agency costs are substitutes for each other: Any reallocation of control rights between investors and managers decreases one type of cost but increases the other.’ (Goshen & Squire, 2017, 771). Depending on the relative expertise of principals and agents, and many other factors (such as characteristics of the sector in which the firm is active), an optimal arrangement may be anywhere along this spectrum (Goshen & Squire, 2017, 797). Recognizing these two types of costs and their trade-offs, gives us a neat theorization of the trade-off between responsiveness (generating principal costs) and independence (generating agency costs) along the spectrum of the control dimension.

These trade-offs exist when the principal group is relatively homogenous or when it is more heterogenous (i.e., it is independent from the constituency dimension). But when the principal group is more heterogenous, then a second source of principal costs arises. Goshen and Squire call these ‘conflict costs’: the costs arising due to conflicts between principals with different interests, when each has a share of control and they have to make decisions collectively. Henry Hansmann’s category of ‘collective decision-making costs’ can be seen as a prominent example of such conflict costs.^20^ Delegating control to an agent decreases these principal costs, but raises agency costs. Conflict costs because of heterogeneity, one could say, can be either converted into principal costs or agency costs (or a combination of both). Governance for purpose, by raising the heterogeneity of stakeholders who are to be considered principals of the firm, has to deal with this predicament.

### Option 1: shareholder-responsive boards

The first policy option in the trilemma, at the bottom of the triangle, is to have boards act as agents of the shareholders as the only group of principals.^21^ This combines minimal principal costs with minimal agency costs. The idea here is that shareholder-oriented boards have low principal costs by their very nature, since they are oriented towards a single objective: shareholder value maximization.^22^ This is not to say that strategic decision-making about what would satisfy this objective cannot be incredibly complicated. But the additional complications (hence costs) of having to compromise between various heterogenous principal groups are absent. This allows shareholders to focus on principles and policies minimizing agency costs, following the precepts mentioned in Sect. "Two Dimensions of Corporate Governance", which discipline boards to follow their shareholders’ interests.

Shareholder value maximization is efficient for society as well, Jensen argued, since it allows the firm to concentrate on a single objective. There is one condition for this congruity between shareholder and societal value maximization to hold: externalities must be absent (Jensen, 2002, 239). The purpose-paradigm comes in here. The concern for a social purpose is a concern for the interests of non-shareholding stakeholders, which get harmed as a result of a shareholder-oriented governance structure. The idea is that externalities do not just ‘exist’ by nature; they are imposed on the parties which are harmed. How does this happen? Colin Mayer summarizes an established line of critique when he argues that shareholders are in an asymmetric position with respect to the corporation, given their limited liability. They do not suffer the full downside risks, while they do benefit from the full upside. As a consequence, they have a preference for risky investments, which impose externalities on other stakeholders (Mayer, 2013, 36–39; see also Ireland, 2010; Mehrotra & Morck, 2017). This point can also be put in the language of agency theory. In their handbook on corporate law, Kraakman, Armour and Davis formulate it as follows: ‘governance arrangements that reduce managerial agency costs by empowering the shareholder majority are likely to exacerbate the agency problems faced by minority shareholders and non-shareholders at the hands of controlling shareholders.’ (Kraakman et al., 2017, 79).

If one wants to resolve stakeholder externalities at the level of the corporation, one needs to move away from shareholder value maximization, towards a corporate purpose which incorporates the interests of all stakeholders.^23^ However, there are two routes for doing so.

### Option 2: independent boards

In the trilemma, the route leftwards is to establish an independent board, which acts on behalf of ‘the corporation’ as a principal. This is an abstraction referring to the purpose of the corporation, as laid down in its charter. Abstractions require interpretation if one wants to act on them, so this leaves the board in the position to interpret the purpose’s implications for corporate strategy.

If this works, two out of the three values in the trilemma are satisfied. First, stakeholder externalities are minimized, assuming that the independent board’s interpretation of the corporate purpose adequately balances all their interests. Second, this is done at low principal costs, since the independent board can take decisions without the costly decision-making process between various principal groups that characterizes stakeholder-responsive boards (see hereafter). The latter point was made by one of the most influential corporate governance theories developed as an alternative to agency theory: team production theory. Blair and Stout argued that firms are best seen as cooperative ventures consisting of various stakeholders (teams), each of which bring specialized assets to the firm. Each of these stakeholder groups needs to get a part of the cooperative surplus. They have an interest in trying to get as large a slice of the surplus as possible. Giving the teams a say over the decision about the surplus is counterproductive, however. If the surplus is decided ex ante, teams will have an incentive to shirk. If the surplus is decided ex post, teams will engage in costly ‘haggling’ over the surplus (Blair & Stout, 1999, 272). To solve this problem, a ‘mediating hierarch’ is needed, who decides the distribution of the surplus. This is the independent board.

The drawback of such an independent board, agency-theory-inspired critics of Blair and Stout have been quick to point out, is that it leads to unaccountable decision-making. Blair and Stout themselves liken their mediating hierarch in a footnote to the solution Thomas Hobbes proposed in political theory, to deal with warring individuals in a state of nature (Blair & Stout, 1999, 274). But John Locke and countless others after him have criticized Hobbes for thinking that ‘men are so foolish that they take care to avoid what mischiefs may be done them by pole-cats, or foxes, but are content, nay think it safety, to be devoured by lions’ (Locke, 2003, 140).^24^ Unbound mediating hierarchs are likely to behave as unaccountable autocrats, taking good care of their own interests while neglecting the corporate interest. In modern terminology, agency costs are high.^25^

### Option 3: stakeholder-responsive boards

As a solution to this problem, one may go for the route rightwards in the trilemma and combine the internalization of stakeholder externalities with a focus on minimizing agency costs. The way to do so is to make the board responsive to the wider set of principal groups.

This is anathema to agency theory as it is presented in the corporate governance literature. Agency theorists usually hold that one cannot hold a board effectively accountable to multiple objectives. In their interpretation, a multiplicity of objectives gives carte blanche to the board to argue that any particular balancing of these interests was in the corporate interest (Jensen, 2002, 242; Heath, 2014, 81; L. Bebchuk & Tallarita, 2020, 53–56).^26^ However, such unaccountability arguments only work against (purely) independent boards. But in politics, in multi-party-systems of government, we regularly see that a government is held to account by two, three or four parties joining in a coalition. These parties closely scrutinize whether the compromises struck by the government faithfully track the balance of forces between the coalition parties. This requires a lot of work, of going back-and-forth between the government and the parliamentary factions of the coalition.^27^ In other words, principal costs are high – exactly as our trilemma would predict. But this is different from saying agency costs are high; that diagnosis misses the mark for this policy option. So the fact that agency theorists often claim stakeholder theory generates high agency costs, betrays the fact they are not actually thinking of a process in which stakeholders take an active role as principals, as sketched here. They are rather thinking of the independent board option discussed earlier.

Hayden and Bodie come to a similar diagnosis. They argue that ‘board primacy theorists’ like Blair and Stout are too quick in responding to heterogeneity by bringing in an independent board. While recognizing that more heterogeneity ‘would, at some level, result in less harmonious decision-making’ (Hayden & Bodie, 2020, 133), they argue that ‘Consensus among the voters or the board members (..) is overrated, especially when that consensus is bought at the price of excluding from the process those with differing views. And claims about the efficiency of the decision-making process all depend upon what is being maximized, which is sometimes at the heart of the disagreement.’ (ibid., 134). They use this argument as a steppingstone to argue in favor of a third position, which neither falls back to shareholder primacy, nor moves to board independence; namely to enlarge the franchise to other stakeholders (only workers, in their view). These are exactly the three positions in the trilemma.

This discussion shows, in my view, how the trilemma often risks being reduced to a dilemma: a move to ‘stakeholderism’ or ‘purpose’ (a move along the constituency dimension) is associated with a move to managerial independence and unaccountability (along the control dimension). Another sort of reduction happens when the trade-off between principal and agency costs is obscured. For example, Kraakman et al. in their handbook write: ‘the more difficult it is for principals to coordinate on a single set of goals for the agent, the harder it is to ensure that the agent does the “right” thing. Coordination costs as between principals thereby exacerbate agency problems’ (Kraakman et al., 2017, 30). Later this is explained as follows: ‘Coordination costs between principals will make it more difficult for them either to *monitor* the agent so as to determine the appropriateness of her actions, or to *decide* whether, and how, to take action to sanction nonperformance.’ (Kraakman et al., 2017, 31–32).

Passages such as these seem to implicitly compare a situation with a relatively homogenous principal group with a situation with a more heterogenous principal group, concluding that in the latter situation ‘coordination costs (…) exacerbate agency problems.’ This move reduces the trilemma to a dilemma: for or against acceptance of more heterogeneity. But a more accurate representation of the situation is that the *combination* of both costs rises as a consequence of the underlying common cause (increased heterogeneity). What gets obscured is that we face a trade-off *between* these two types of costs, *within* this situation of increased heterogeneity. The comparison between ‘option 2’ and ‘option 3’ in the trilemma is about this: two versions of the situation, in which the principal group displays a higher level of heterogeneity.

In conclusion, we need to remain aware of all three options in the trilemma, and the tensions between them, and avoid reductions. My hope is that the trilemma serves as a disciplining tool, so that we are able to disentangle both dimensions of corporate governance and then analyze the combinations of options this yields.^28^ Only then can we discuss the trade-offs for what they are.

### The trilemma’s wider applicability: ‘pre-purpose’ corporate governance

This finishes the overview of the trilemma. In connection to the history of corporate governance, one additional feature of this trilemma should be noted, however. So far, I have presented it as a trilemma which sheds light on the move to purpose-governance. But the shareholder-stakeholder debate has not always been at the center of corporate governance. Rather, at the center was (and for some still is) the tension between majority and minority shareholders. The crucial question in that context is how to protect minority shareholders from a tyranny of the majority. In the corporate purpose debate, shareholders are represented as a homogenous group, at least relatively homogenous compared to other stakeholder groups. But shareholders themselves are – or at least can be, depending on which parties hold shares – themselves a very heterogenous group as well. And to the extent that shareholders have heterogenous preferences (about time horizons, investment strategies, etc.), the trilemma also holds for situations marked by majority-minority shareholder conflicts. The first option then is to solve these conflicts through the fictitious construction of shareholder wealth maximization as a homogenizing norm (Greenwood, 1996). This will generate externalities, namely those (minority) shareholder interests which are squeezed out, under this norm. The second option is to resolve these conflicts through corporate politics, where shareholders can try to form coalitions. Third, an independent board can be called upon to resolve these conflicts, mediating between the various shareholder groups.^29^ The corporate governance trilemma is hence more general. It applies to all situations in which heterogeneity between principals crosses some threshold level of significance, whether these principals are (only) shareholders or (in addition) non-shareholding stakeholder groups.

This wider applicability of the trilemma also helps us to position one policy option in discussions of purpose-driven corporate governance that I haven’t mentioned so far. This option is to keep corporate governance as it is, but ask (expect, nudge, etc.) shareholders to represent the interests of stakeholders. The heterogeneity of shareholders is usually portrayed as a matter of diverging financial interests (e.g., long versus short time horizons of investment). However, some shareholders may have a wider view of their welfare, which goes beyond their own financial wealth. They may start to act as representatives (agents!) of these other stakeholders, whose welfare becomes part of their preferences (Hart & Zingales, 2017). This move maintains ‘shareholder primacy’ (i.e., ‘shareholder-responsiveness’ in my framework), but does away with shareholder wealth maximization as its objective.

In practice, the ESG-movement is built on the acknowledgment that shareholders should care about stakeholder-interests. In the language of agency theory, this can be described as a double-layered agency construction, in which shareholders are intermediaries between stakeholders (whose agents they are) and boards (whose principals they are)(Raelin & Bondy, 2013). If one wants to represent this option in the purpose-trilemma, then it would be a subcategory of ‘stakeholder-responsive boards’, where stakeholders’ interests are represented not directly (through their own voice) but indirectly, via the voice of shareholders. Whether this is a promising route towards more effective purpose-governance I leave out of consideration.

## The Independence-Responsiveness Trade-off

In this final section I want to move beyond the analytical contribution of constructing the trilemma to considerations of a more explicitly normative kind. The question is which position purpose-advocates *should* choose in the trilemma. I first reflect on this question in the framework of economic theory and then argue that political theory can help us here as well.

Most obviously, purpose-advocates do not opt for the option of shareholder-responsive boards. But it is worth specifying why. As we saw, this position leaves (large) stakeholder externalities uninternalized.^30^ But as we have also seen, the other positions create inefficiencies of their own, i.e., high agency costs or principal costs. Hence using an economic framework, a purpose-advocate must show that the costs of these externalities exceed the efficiency benefits on these other dimensions.^31^ Purpose-advocates make this argument, for example, when they argue that at the current stage of development of the more affluent economies, the relative scarcity of financial capital compared to non-financial capitals has declined. In other words, at this stage of development, financial capital has become relatively abundant. Natural and social capital however, have become relatively scarce, and set-backs on these capitals to the benefit of financial capital therefore are now lowering, not increasing, aggregate wealth in society (Mayer 2018). Meanwhile, the fact that the market mechanism punishes corporations with high agency and/or principal costs with a competitive disadvantage, while (by definition) not punishing corporations with high externalities, explains why this overall inefficiency can persist.

The next step in their argument is that internalization of a particular externality at the level of the corporation is more efficient than internalization by external, government-mandated regulation. The argument here relies on the presence of government failure in regulating externalities. Shareholder theorists have pushed back against purpose-advocates, by arguing that government should regulate externalities, not corporations themselves (Bebchuk & Tallarita, 2020). Stakeholder theorists have argued this ignores widespread government failure. The causes for such failure are varied: from epistemic problems with respect to the ability of government institutions to gather all relevant firm-level information to regulate adequately; to problems of regulatory capture where firms influence policy-making processes to their own advantage. Also, the context of the globalized economy generates large-scale externalities that states are incapable of resolving on their own (Stoelhorst & Vishwanathan, 2024). Here let’s assume that – at least for certain types of externalities – this is a convincing diagnosis. Hence the combination of market failure and government failure points to resolving – at least some – externalities at the level of the corporation itself. For those externalities, the key corporate governance question becomes whether to internalize them via stakeholder-responsive or independent boards.^32^

This choice can be made by comparing the economic costs of each of the values and maximizing the sum total.^33^ This would require that all costs can be predicted and monetized, which is far from self-evident (e.g., how to calculate unpriced externalities? And what costs to attach to ‘suboptimal decisions’ due to more complex decision-making? What is the baseline against which to compare these?). I will not pursue a criticism of the reductivism of economic method here, however. Regardless of the merits or limits of economic methods, I want to mobilize some considerations from democratic theory, to argue in favor of accepting, as the best option, a point somewhere in between the ‘independent board’ option and the ‘stakeholder-responsive’ option, a balance or mix between both extremes (see also Claassen, 2025, 10–11). The central point was made eloquently by Pitkin who said about the delegate-trustee spectrum (Sect. "Two Dimensions of Corporate Governance" above): ‘If a state of affairs deviates too much in one direction or another, we shall say that it is no longer representation at all (he is simply an oligarch; he is simply a tool)’ (Pitkin, 1967, 166). Let’s unpack both sides of this statement.

On the one hand, adequate forms of representation cannot be purely delegate-based. This makes the representative into a mere ‘tool’ in the hand of the represented. This argument has been the foundation for so-called *constructivist* theories of representation. A representative always makes a representation (noun), i.e., a certain picture or portrayal of the interests of the person(s) they represent. Constructivists argue that these are not two separate things, however: ‘the identity, interests, or preferences of the represented are not given prior to representation but shaped through being represented.’ (Fossen, 2019, 824). Representations by members of a board or government co-construct the interests of the constituencies that they purport to represent. In the corporate context, constructivism can serve as a healthy warning against all-too-reified conceptions of corporate purpose. Just stating a purpose in a corporate charter is not going to set any meaningful limits to the decisions of the corporation, in the absence of an interpretation of that purpose by the representative, the corporate board. To create this interpretation, a certain freedom from the instructions of particular constituencies seems to be needed.

The underlying picture of decision-making is one that rejects purely aggregative models, in which members’ preferences serve as force vectors, which when combined suggest a certain direction – the action to be undertaken is the action which the aggregate vector points to. Such a mechanistic view of decision-making depicts compromises between two constituency groups as arithmetic positions in between the two groups’ original positions, depending on strength and intensity of the preferences and sizes of the groups. Such a picture of decision-making misses the judgments needed to systematize (potentially incommensurable) preferences into a coherent plan of action, which can take the group as a whole on a path which offers it chances of success in the wider, outside environment (here: market competition).^34^ The corporate purpose (and board decision-making more generally) is the outcome of such judgments, this irreducible systematization, which may be more than a simple sum of the parts. To find such creative compromises with survival potential, would seem to require independence of the decision-makers.

However, this is only one side of the coin of Pitkin’s statement. On the other hand, representatives whose decisions are completely severed from any feedback by and accountability to their constituencies risk becoming – in her terms – ‘oligarchs.’ (or as we saw earlier, in the context of Blair and Stout’s team production theory, Hobbesian autocrats). The material from which a representative must draw a particular representation of the ‘corporate interest’, are the preferences and interests of the constituencies, and hence some link to them needs to be maintained. One of the reasons for this is epistemic: the representative must have sufficient knowledge about these interests, which constituencies themselves are best positioned to provide. Another reason is political: as agency theorists stress, giving too much power without accountability to representatives enables them to prioritize their own interests, exploiting those whom they are meant to represent. Restraining the freedom necessary for representatives to create their representations doesn’t mean giving up on the constructivist nature of representation. Rather, it acknowledges that the construction is a co-production of the representer and the represented – a point in between the pure delegate model and the pure trustee model of representation.

Taken together, then, it seems that good corporate governance needs both a measure of board independence (to ensure coherence in purpose and creativity), and a measure of co-decision-making powers by stakeholders (to ensure maximal input of information and protect them against exploitation), hence a governance structure which combines elements of both. This is important, given the tendency in the literature (see discussion of options 2 and 3 above) to plead for either (full) ‘board independence’ or (full) ‘board responsiveness’, which would imply that the best structure approximates as closely as possible one of these ideal types. Once we reject such purity, and think in terms of a balance, the further question then becomes where and how exactly to strike this balance, in terms of procedures for consultation, participation and voting. This is the task of designing a corporate governance regime for purpose-driven business. The specifics of such a regime will be dependent on legal, sectoral, cultural and other contextual variables, and cannot, I think, be formulated in the abstract. But it is helpful to look into an illustration to get an idea of how the conflicting demands of responsiveness and independence play out, in the context of purpose-driven business. To this end, I now turn to one particularly high-profile case of the challenges for purpose-governance, i.e., the 2023 corporate governance troubles of OpenAI.

OpenAI was founded in 2018 to compete with highly commercial big tech companies in developing new AI models. Its governance structure is peculiar and can be interpreted as an instance of steward ownership.^35^ OpenAI states as its purpose: ‘building safe and beneficial artificial general intelligence for the benefit of humanity.’^36^ Simplifying somewhat, the founding entity is a public charity (OpenAI Non-profit) which holds the control rights over the main operating entity (OpenAI Global, LLC).^37^ The purpose has implications for the board’s fiduciary duties, which are to humanity as a whole: ‘The Nonprofit’s principal beneficiary is humanity, not OpenAI investors.’^38^ The board is self-electing its members with a simple majority. Finally, OpenAI Global shares were originally only held by OpenAI Non-profit. Later, capped dividend rights (without voting rights) were given to employees and to Microsoft, amounting to a minority of all outstanding stock.^39^ Microsoft made important investments in OpenAI and received contractual benefits in return.

Late 2023, in a majority vote the four board members of OpenAI fired the other two board members, Sam Altman, the CEO, and Greg Brockman. In the context of heated public debates about the existential risks of AI, this decision was generally interpreted as inspired by the board’s wish to put safety-first in the development of the newest AI models, whereas Altman wanted to give more weight to speed, and commercial interests. A massive uproar of employees ensued, as well as a move by Microsoft to hire Altman and Brockman. Rumors spread that Microsoft was also willing to hire large numbers of employees, basically re-creating OpenAI within Microsoft. As a result, within a week the decision was reversed. Three new directors were installed to replace the four original directors, and Altman and Brockman were reinstated as executives.

In light of the discussion in this paper, how to interpret this ‘OpenAI saga’? The original board members were – to their own judgments – acting on their interpretation of the purpose, as they should since this is their fiduciary duty. They judged Altman and Brockman’s positioning of the company to threaten the purpose and acted accordingly. The problem is that they did so in independence of the other stakeholders. Part of the criticism was procedural: the board’s decision was criticized for being insufficiently prepared by consultations with relevant stakeholders (employees and Microsoft). But the board was just doing what it was supposed to do according to the governance structure that was set up: trying – in its independent judgment – to track the interests of OpenAI’s only beneficiary (humanity); neglecting the interests of stakeholders who are not specifically targeted in the purpose. Therefore I want to argue that ultimately the events were the all-to-be expected result of two more structural problems underlying the chosen purpose and governance structure.

On the constituency dimension, the purpose statement included only one beneficiary group, i.e., humanity, and did so to the explicit exclusion of investors (and implicitly, also any other group). Here, I interpret ‘humanity’ as the users of OpenAI products. Strictly speaking, of course employees and Microsoft are part of humanity as well, but ‘humanity’ as the beneficiary of the development of AI, seems about those who benefit from it as users. So I will refer to this beneficiary group here as “AI users.” The other stakeholders, employees and shareholders such as Microsoft, did not have principal/beneficiary status, nor did they have control rights. When faced with a conflict between both groups (as was the case here, if we follow the media reports), the board chose to prioritize humanity’s interests (on its interpretation) over that of the other stakeholders. But it did so in a way that underestimated the company’s de facto dependence on its employees and on Microsoft. Hence the tumult and eventual reversal of the decision. Without them, the company would end being an empty shell. This suggests that the purpose statement and governance structure should have included them as well. Excluding them from the governance structure will tend to undermine the support for their cooperative relation to the enterprise (especially in a context where they have outside options).

Second, in terms of the control dimension, OpenAI’s structure leans towards the extreme independence pole of the spectrum (which we saw above Pitkin identified with oligarchy). Strikingly, there is an absence of responsiveness to *both* sides in the dispute. In this case, this lack of responsiveness was exposed only by one side: the employees and Microsoft. The governance structure was dysfunctional by leaning too heavily towards the independence side on the control dimension. A more responsive governance structure is warranted.^40^ In the aftermath, OpenAI worked on this, for one side of the dispute. Microsoft managed to get an observer’s seat at the board, to be better informed of the board’s deliberations and decisions. This increases responsiveness towards one side. No move was made to counterbalance this, however, by increasing the responsiveness to AI users (‘humanity’). The latter do not have any control rights towards the OpenAI Nonprofit. Hence there is no possibility to give any feedback or organize any process of deliberation, criticism or accountability, for the OpenAI Nonprofit’s board’s particular interpretation ofwhat is to count as the interests of humanity. Of course, there’s a tough question about whether this could be organized, given the challenging nature of representing ‘humanity’ as a whole. But perhaps OpenAI could have made use of trustees representing humanity.^41^ Or OpenAI could have restated ‘humanity’ in conventional stakeholder terms as the ‘users/consumers’ of its products and allow for a representation of users.

From the OpenAI case, we can learn at least two things about corporate governance ‘for purpose.’ First, a firm which defines a purpose and adopts a corresponding board mandate (fiduciary duty) which focuses only one stakeholder group, to the exclusion of all others, risks conflicts with the neglected stakeholder groups. This suggests adopting a purpose statement which does justice to the full stakeholder spectrum (constituency continuum) actually involved in the production process that the firm aims at. Second, on the control dimension, as the trilemma suggests, a fully independent boards risks high agency costs (and hence dissatisfaction of the principals). A more responsive structure on both sides would increase principal decision-making costs (OpenAI’s controversial board decision was certainly low on decision-making costs). But it would decrease the agency costs involved, as the trilemma also stresses. The conflicting interests between the two principal groups won’t thereby vanish, but one would expect that they would be more carefully mediated in the board’s actions.

## Conclusion

The paper has offered a trilemma model which aims to be helpful in moving forward the corporate governance discussion in the context of purposeful business. It has done so by distinguishing two separate dimensions in governance (constituency and control dimensions). The main question becomes what a broadening of the constituency basis of a corporation (from shareholders to stakeholders) means for the control dimension (independence versus responsiveness). The analysis shows the trade-off between the various values involved (principal costs, agency costs and externalities). In the final part, I argued a balance between independence and responsiveness must be struck. This was illustrated with the example of OpenAI, which shows the dangers of leaning too much towards pure independence. This example also vividly reminds us that a larger constituency basis inevitably leads to the need to reconcile more (conflicting) interests – which is the price to pay for corporate governance to the benefit of societal purposes.

## Data Availability

Not applicable.

## Footnotes

1 https://www.businessroundtable.org/business-roundtable-redefines-the-purpose-of-a-corporation-to-promote-an-economy-that-serves-all-americans. Later, many observers noted that in practice, the corporations part of the BRT hadn’t changed their business practices, adding to widespread cynicism about ‘purposewashing.’ (L. A. Bebchuk and Tallarita 2022).

2 I will simply speak of ‘the board’ in this paper as the organ leading the corporation, both ignoring the complication of dual-board systems and the distinction between executive and non-executive officers in single-board systems.

3 Eward Rock has distinguished the following dimensions in the debate about purpose: 1) a legal debate about the best interpretation of the law as it is: what is the ‘best “theory” of the corporate form’ (p. 7); 2) a finance question about ‘how firms are actually managed’ (p. 17); 3) a management debate about whether a focus on share prices as a ‘single metric’ leads to more long term firm value or not (p. 20); and 4) a political debate over the ‘social role of large corporations’, and the obligations imposed on them (p. 6). Put in this way, this paper is about the fourth question, since this is the only one which is not confined to corporate law as it is (debate 1) or how the firm is managed (debate 2) or should be managed given the objective long term firm value in financial terms (debate 3). Following Mayer and others, the paper adresses the normative question what the law should be, and how firms should be managed if the purpose would go beyond long term firm value, to include externalities.

4 Externalities whose internalization is beneficial to shareholders, can be resolved by ‘shared value’ business strategies (Porter and Kramer 2011). The purpose-paradigm presupposes that there are (a lot of) externalities for which this is not the case. For a critique of Porter and Kramer, see (Crane et al., 2014); see also the critique of ‘enlightened shareholder value’ in (Bebchuk, Kastiel, and Tallarita 2022).

5 Although less explicitly, one can construe two similar wings in stakeholder theory. Moriarty argues there has been a (largely tacit) shift between earlier and later stakeholder theorists, with respect to their stance to the question of democratization. See (Moriarty 2014).

6 Strine argues for adopting co-determination laws in the US in (Strine Jr, Kovvali, and Williams 2022).

7 There is debate on whether the terminology of ‘membership’ is appropriate in the context of business corporations (Ciepley 2020; Mansell and Sison 2020). Here I use the term ‘member’ broadly, but without taking a stance in these disputes.

8 This is controversial, since some lawyers argue that the law (for example in the US) should be interpreted as putting the board in the position of agents of the corporation itself (Lan and Heracleous 2010; Stout 2012). This would already point to the purpose-paradigm.

9 Independence doesn’t mean ‘freedom’ in that the board can posit any goal as the corporate goal (including completely self-serving actions). It is legally bound to take the interests of their principals (or the corporate purpose, as a summary statement of the combination of the interests of the corporate members) as their objective. The point is that an independent board has discretionary power in judging how to fulfil this interest/purpose, i.e., it is unchecked (in the limit case of ‘pure independence’) by governance mechanisms which would empower corporate constituencies (or outsiders, such as the courts) to influence or overrule its decisions.

10 A separate terminology from fiduciary law describes boards/agents as ‘fiduciaries’ and shareholders/principals as ‘beneficiaries’. The principal-agent relation can be seen as a species of fiduciary relationships. Some argue however, that the two are opposed legal models (Clark 1985). I remain agnostic here, but see my adoption of the fiduciary model in (Claassen 2024).

11 See (Goshen and Squire 2017)’s theory of principal costs for elaboration of this point.

12 These are all mechanisms of ‘voice’. Agency theorists have additionally favored ‘exit’ mechanisms to impose board-responsiveness: the threat of hostile take-overs, i.e., ‘the market for corporate control’ (using Hirschmann’s classical voice/exit distinction, although we should note that the pressure on boards here is exercised not just by exiting shareholders, but also by entering shareholders who use ‘voice’ to express their will). I here focus on responsiveness through mechanisms of ‘voice’, in line with classical political theory.

13 This is the ‘pure’ arrangement. In an ‘impure’ arrangement, some shares with profit rights (but without voting rights) are given to outside investors with commercial interests. The large foundation-owned companies in Denmark (Novo Nordisk, Maerk, Carlsberg) have a minority of economic rights listed publicly.

14 Steward-ownership is a slightly wider category than foundation ownership, since it also allows for a committee of persons within the company to fulfill the role of stewards (so that one need not set up a separate foundation for this). For press coverage of steward-ownership, see https://www.newyorker.com/business/currency/can-companies-force-themselves-to-do-good.

15 https://www.theguardian.com/us-news/2022/sep/14/patagonias-billionaire-owner-gives-away-company-to-fight-climate-crisis-yvon-chouinard. For an analysis of Patagonia by the Purpose Foundation, see https://medium.com/@purpose_network/the-patagonia-structure-in-the-context-of-steward-ownership-e9db3d260dc6.

16 One divergence is that some countries which have adopted separate enterprise foundation statutes specify that foundation directors must be economically and socially independent from the founders of the company and from the company they supervise (Sanders and Thomsen 2023, 233–36). An empirical study on the governance of these foundation-owned companies also suggests that their success is linked to the independence of the foundation board vis-à-vis the company board (Hansmann and Thomsen 2021, 224).(note that ‘independence’ in this context is not about the empowerment of boards vis-à-vis their controllers, but about the sociological/psychological independence of the controllers, strengthening their position to the board!).

17 If such a responsiveness to workers is created, then steward-owned companies’ control structure comes close to yet another corporate form, that of worker cooperatives (but without the economic rights being held by the workers).

18 When combining the two dimensions, we get four ideal type positions (a two-by-two matrix with independence versus responsiveness, and homogeneity versus heterogeneity). The trilemma excludes the position combining homogeneity with independence, e.g., Bainbridge’s ‘director primacy’ model, combining board independence with shareholder wealth maximization as the goal (Bainbridge 2003). because the reason for this is the focus is here on the purpose-paradigm, i.e., those positions implying a shift to heterogeneity. Independence may be warranted for other reasons, in particular because principals lack competence to make decisions themselves, and/or their wish to save their personal time/energy in decision-making. But these other reasons (and the level of independence, i.e., the level of transfer of power from principals to agents to which they give rise) are here taken as the baseline level against which the trilemma plays out. (see the discussion of Goshen and Squire in the main text).

19 With Michael Bennett, I introduced another trilemma in (Bennett and Claassen 2022). That trilemma focused on the trade-off between ‘powerful firms’ (similar to the ‘shareholder-responsive boards’ option here), ‘accountable firms’ (here divided into two options, ‘independent boards’ and ‘stakeholder-responsive boards’) and ‘competitive firms’ (here ignored). Bennett & Claassen’s trilemma studies the trade-offs between the standard shareholder-controlled firm and two alternative options creating a ‘power balance’ between shareholders and stakeholders, one *internal* to the corporation’s governance structure (accountability), the other *external* to the corporation (competition); the trilemma here moves to zoom in on two different internal paths to power balance. The values addressed in the dilemma change accordingly.

20 Hansmann rightly distinguishes these from agency costs, but defines them as the surplus costs of heterogeneity in the principal group (Hansmann 1996, 40). The Goshen and Squire framework is more encompassing since it also includes principal and agency costs as a result of incompetence, in conditions of complete homogeneity of interests between principals.

21 Most often we think of outside investors as the relevant shareholders. As Hansmann pointed out, workers, consumers or others can serve as the relevant ownership group as well, and do so in some firms such as worker or consumer cooperatives (Hansmann 1996). So the option here could also be called ‘ownership-responsive boards’ (using Hansmann’s terminology; and bracketing the debate about whether shareholders should be termed ‘owners’ at all, given how their bundle of rights differs from that of a standard owner of a material object).

22 In presenting option 1 in the trilemma, I keep things simple and closely associate the concept of shareholder-responsiveness in corporate governance (also referred to as ‘shareholder primacy’) with shareholder wealth maximization as the objective. But see the end of this section, where I complicate this association. See (Smith and Rönnegard 2016) for a discussion of the relation between these two concepts.

23 Critics of the purpose-paradigm often argue externalities should be resolved through external government regulation (Bebchuk and Tallarita 2020). I come back to this in Sect. "The Independence-Responsiveness Trade-off". One can also argue that besides government regulation and corporate governance, ethical restraint is a third option (Heath 2014; 2023). I leave the latter out of consideration, since the moral sense (conscience) of corporate leaders on its own (i.e., in the absence of supporting institutional/governance mechanisms) is likely to have some, but arguably not enough force to fully resolve the externalities in question, in a field with strong counteracting incentives. One can read this paper as focusing on whatever level of corporate externalities remain a feature of our social world, ‘after’ moral sense has done its salutary work.

24 I thank Michael Bennett for reminding me of Locke’s words as suitable for this context.

25 Blair and Stout think this problem can be mitigated by giving the hierarch ‘a nominal share of the team’s output.’ (Blair and Stout 1999, 274).

26 This remains a highly influential argument. However, as mentioned earlier, there is nothing inherent in agency theory prohibiting the acknowledgment of multiple principals, and similarly, multiple objectives. Indeed, some agency theorists, such as Jensen, seem to have changed their mind on this topic; see (Dierksmeier 2020).

27 I here make use of multi-party coalitions in a discussion about assigning control rights to stakeholders. In parallel, Heath has used the possibility of multi-party coalitions to throw a critical light on Hansmann’s argument about the most effective assignment of ownership rights (Heath 2014, 132–36).

28 See also note 18, for how a full discussion of both dimensions would yield a two-by-two matrix with four ideal type options.

29 Indeed, Hayden and Bodie argue that it was shareholder heterogeneity that led to the rise of board primacy theories, see (Hayden and Bodie 2020, 122).

30 This formulation leaves open the viability of internalization for some externalities through market pressures; e.g., because consumers’ concerns act as a restraining force on managers (reputation effects in markets); or in situations where there is ‘shared value’; see note 4 above. The issue here is those externalities for which this doesn’t work.

31 Here I assume a simple maximizing economic framework across all three factors. Different distributive rules (inspired by non-maximizing theories of justice) would attach different weights on all three factors; see e.g., my overview of rules in chapter 4 of (Claassen 2018). This is beyond the scope of my presentation. But such non-maximizing rules (e.g., sufficientarianism) could protect against environmental and social harms even in societies at lower levels of financial wealth.

32 As this sentence implies, I concentrate in the remainder of this section on the choice on the control dimension, given a certain degree of internationalization (i.e., broadening on the constituency dimension). This implies that I offer in the remainder of this section only a partial analysis, which works with the *ceteris paribus* clause that any choice on the control dimension would not be affected by choices on the constituency dimension. This is of course unlikely: for example, the nature of the stakeholders included on the constituency dimension may make a difference in the mix between independence and responsiveness that is optimal; similarly, the extent to which these stakeholders’ interests are included may matter for the mix between independence and responsiveness that is optimal; etc. As stated at the beginning of Sect. "The Corporate Governance Trilemma", even purpose-advocates do have to navigate the full trilemmatic space. The reason that I here abstract from these interdependencies, which will arise in practice, is merely to keep the analysis tractable. I thank one of the reviewers’ for pointing to this complication.

33 Stoelhorst and Vishwanathan (2024) offer an example, giving a comparative efficiency analysis of various forms of corporate governance, shareholder- and stakeholder based.

34 For a similar recognition of the incommensurability of plural stakeholder preferences, see (Mitchell et al., 2016). To deal with this pluralism, they emphasize the need to construct ‘intracorporate stakeholder markets.’ (see pp. 265–266). My emphasis here is on the interpretative task of making judgments, which combine these preferences into one corporate purpose, and its interpretation in corporate action.

35 For an analysis in the light of steward ownership, see two blogposts: https://medium.com/@purpose_network/openai-in-the-context-of-steward-ownership-blog-post-1-on-the-openai-saga-dd07fea9eecd and https://medium.com/@purpose_network/bringing-steward-ownership-to-life-while-playing-on-the-field-of-capitalism-blog-post-2-on-the-81eb973948af

36 See https://openai.com/our-structure.

37 I abstract here from the intermediate entities involved, as well as the partnership nature of the structure. For all the details, see https://medium.com/@purpose_network/openai-in-the-context-of-steward-ownership-blog-post-1-on-the-openai-saga-dd07fea9eecd

38 See https://openai.com/our-structure.

39 This is the ‘impure model’ of steward ownership, see note 13.

40 In his analysis of the case, Tallarita comes to a similar conclusion: ‘Socially-oriented governance structures should not be content with independence from executives and investors. They should also set up mechanisms that encourage directors to purse the social goal and take them to account. Corporate planners should experiment with methods that allow outside scrutiny of board decisions, with incentives for socially-oriented decision-making, and with creative forms of accountability for board members.’ (Tallarita 2023).

41 Some companies experiment with representing non-human nature in the governance structure by appointing trustees. See e.g., https://nieuweinstituut.nl/en/projects/zoop/zoop-model. Also there’s the movement for granting legal personhood to nature which makes use of trustees (Sanders 2018). If non-human nature can be represented this way, so can humanity.

## References

[CR2] Anderson, E. (2017). *Private government. How employers rule our lives (and why we don’t talk about it)*. Princeton University Press.

[CR3] Aytac, U. (2025). What is the point of social media? Corporate purpose and digital democratization. *Philosophy & Technology,**38*, 26. 10.1007/s13347-025-00855-y 39990541 10.1007/s13347-025-00855-y PMC11842518

[CR4] Bainbridge, S. M. (2003). Director Primacy: The means and ends of corporate governance. *Northwestern University Law Review*. 10.4324/9781315574288-7

[CR5] Bebchuk, Lucian A., Kobi Kastiel, and Roberto Tallarita. 2022. “Does Enlightened Shareholder Value Add Value?” *Business Lawyer* 77 (3). 10.2139/ssrn.4065731.

[CR6] Bebchuk, Lucian A., and Roberto Tallarita. 2022. “Will Corporations Deliver Value to All Stakeholders?” *Vanderbilt Law Review* 75 (4). 10.2139/ssrn.3899421.

[CR7] Bebchuk, L., & Tallarita, R. (2020). The illusory promise of stakeholder governance. *Cornell Law Review,**106*(1), 91–178.

[CR8] Bennett, M., & Claassen, R. (2022). The corporate power trilemma. *Journal of Politics,**84*(4), 2094–2106. 10.1086/717851

[CR9] Blair, M., & Stout, L. (1999). A team production theory of corporate law. *Virginia Law Review,**85*(2), 247–328.

[CR10] Blanc, S. (2023). Deliberative democracy and corporate constitutionalism: Considering corporate constitutional courts. *Journal of Business Ethics,**188*, 1–15. 10.1007/s10551-022-05206-x

[CR11] Boeger, N., Burgess, S., & Ellison, J. (2018). Lessons from the Community Interest Company. In N. Boeger & C. Villiers (Eds.), *Shaping the Corporate Landscape: Towards Corporate Reform and Enterprise Diversity* (pp. 347–364). Hart Publishing.

[CR1] British Academy. 2019. “Reforming Business for the 21st Century.” London. https://www.thebritishacademy.ac.uk/publications/reforming-business-21st-century-framework-future-corporation/.

[CR12] Ciepley, D. (2013). Beyond public and private: Toward a political theory of the corporation. *American Political Science Review,**107*(1), 139–158.

[CR13] Ciepley, D. (2017). Is the US government a corporation? The corporate origins of modern constitutionalism. *American Political Science Review,**111*(2), 418–435.

[CR14] Ciepley, D. (2020). The anglo-american misconception of stockholders as ‘owners’ and ‘members’: its origins and consequences. *Journal of Institutional Economics,**16*(5), 623–642.

[CR15] Claassen, R. (2025). “Property as Power. A Theory of Representation.” *Journal of Social Philosophy*. 10.1111/josp.12587.

[CR16] Claassen, R. (2018). *Capabilities in a just society. A theory of navigational agency*. Cambridge University Press.

[CR17] Claassen, R. (2023). Political theories of the business corporation. *Philosophy Compass,**18*(1), e12892. 37033445 10.1111/phc3.12892 PMC10078144

[CR18] Claassen, R. (2024). Wealth creation without domination. A fiduciary theory of corporate power. *Critical Review of International Social and Political Philosophy,**27*(3), 317–338. 10.1080/13698230.2022.2113224 38533485 10.1080/13698230.2022.2113224 PMC10962712

[CR48] Clark Jr., William, and Elizabeth Babson. 2012. “How Benefit Corporations Are Redefining the Purpose of Business Corporation.” *William Mitchell Law Review*. Vol. 38.

[CR19] Clark, R. (1985). Agency Costs versus Fiduciary Duties. In J. Pratt & R. Zeckhauser (Eds.), *Principals and Agents : The Structure of Business* (pp. 55–79). Harvard Business School Press.

[CR20] Crane, A., Palazzo, G., Spence, L. J., & Matten, D. (2014). Contesting the Value of ‘Creating Shared Value.’ *California Management Review*. 10.1525/cmr.2014.56.2.130

[CR21] Davis, G. (2021). Corporate purpose needs democracy. *Journal of Management Studies,**58*(3), 902–913.

[CR22] Dierksmeier, C. (2020). From Jensen to Jensen: Mechanistic management education or humanistic management learning? *Journal of Business Ethics*. 10.1007/s10551-019-04120-z

[CR23] Fama, E. F., & Jensen, M. C. (1983). Agency problems and residual claims. *The Journal of Law & Economics*. 10.1086/467038

[CR24] Ferreras, I. (2017). *Firms as political entities. Saving democracy through economic bicameralism*. Cambridge University Press.

[CR25] Fossen, T. (2019). Constructivism and the logic of political representation. *American Political Science Review,**113*(3), 824–837.

[CR27] Freeman, R. Edward. 1984. *Strategic Management: A Stakeholder Approach*. Harpercollins College.

[CR28] Freeman, E., & Evan, W. (1990). Corporate Governance: A Stakeholder Interpretation. *The Journal of Behavioral Economics,**19*(4), 337–359.

[CR29] Freeman, R. E., Harrison, J., Wicks, A., Parmar, B., & De Colle, S. (2010). *Stakeholder theory. The state of the art*. Cambridge University Press.

[CR30] Frega, Roberto, Lisa Herzog, and Christian Neuhäuser. 2019. “Workplace Democracy—The Recent Debate.” *Philosophy Compass* e12574: 1–11. 10.1111/phc3.12574.

[CR31] González-Ricoy, I. (2022). Little republics: Authority and the political nature of the firm. *Philosophy & Public Affairs,**50*(1), 90–120.

[CR32] González-Ricoy, I., & Magaña, P. (2024). The demos of the democratic firm. *Politics, Philosophy & Economics,**23*(4), 346–367. 10.1177/1470594X241239986

[CR33] Goshen, Z., & Squire, R. (2017). Principal costs: A new theory for corporate law and governance. *Columbia Law Review,**117*(3), 767–830. 10.2139/ssrn.2571739

[CR34] Greenwood, D. (1996). Fictional shareholders: For whom are corporate managers trustees, revisited. *Southern California Law Review,**69*, 1021–1104.

[CR35] Hansmann, H. (1996). *The ownership of enterprise*. The Belknap Press.

[CR36] Hansmann, H., & Thomsen, S. (2021). The governance of foundation-owned firms. *Journal of Legal Analysis,**13*(1), 172. 10.1093/jla/laaa005

[CR37] Harrison, J. S., Phillips, R. A., & Freeman, R. E. (2020). On the 2019 business roundtable ‘statement on the purpose of a corporation.’ *Journal of Management,**46*(7), 1223–1237.

[CR38] Hart, O., & Zingales, L. (2017). Companies should maximize shareholder welfare not market value. *Journal of Law, Finance, and Accounting,**2*, 247–274. 10.1561/108.00000022

[CR39] Hayden, Grant, and Matthew Bodie. 2020. *Reconstructing the Corporation. From Shareholder Primacy to Shared Governance*. Cambridge: Cambridge University Press.

[CR40] Heath. J, 2014. *Morality, Competition and the Firm. The Market Failures Approach to Business Ethics*. Oxford: Oxford University Press.

[CR41] Heath. J, 2023. *Ethics for Capitalists. A Systematic Approach to Business Ethics, Competition, and Market Failure*. Altona, Canada: FriesenPress.

[CR42] Heath, J. (2009). The uses and abuses of agency theory. *Business Ethics Quarterly*. 10.5840/beq200919430

[CR43] Heath, J., Moriarty, J., & Norman, W. (2010). Business ethics and (or as) political philosophy. *Business Ethics Quarterly,**20*(3), 427–452.

[CR44] Henderson, R. (2020). *Reimaging capitalism in a world on fire*. PublicAffairs.

[CR45] Ireland, P. (2010). Limited liability, shareholder rights and the problem of corporate irresponsibility. *Cambridge Journal of Economics,**34*, 837–856.

[CR46] Jensen, M. (2002). Value maximization, stakeholder theory, and the corporate objective function. *Business Ethics Quarterly,**12*(2), 235–256.

[CR47] Jensen, M., & Meckling, W. (1976). Theory of the firm: Managerial behavior, agency costs and ownership structure. *Journal of Financial Economics,**3*, 305–360.

[CR50] Kraakman, R., Armour, J., & Davies, P. (2017). *The Anatomy of Corporate Law. A Comparative and Functional Approach* (3rd ed.). Oxford University Press.

[CR51] Lan, L. L., & Heracleous, L. (2010). Rethinking agency theory: The view from law. *Academy of Management Review*. 10.5465/AMR.2010.48463335

[CR52] Lankoski, L., & Craig Smith, N. (2018). Alternative objective functions for firms. *Organization & Environment,**31*(3), 242–262.

[CR53] Levillain, K., & Segrestin, B. (2019). From primacy to purpose commitment: How emerging profit-with-purpose corporations open new corporate governance avenues. *European Management Journal,**37*, 637–647. 10.1016/j.emj.2019.07.002

[CR54] Locke, John. 2003. *Two Treatises of Government*. *Cambridge Texts in the History of Political Thought*. Cambridge: Cambridge University Press.

[CR55] Mansell, S. (2013). *Capitalism, corporations, and the social contract. A critique of stakeholder theory*. Cambridge University Press.

[CR56] Mansell, S., & Sison, A. (2020). Medieval corporations, membership and the common good: Rethinking the critique of shareholder primacy. *Journal of Institutional Economics,**16*(5), 579–595.

[CR57] ———. 2020. “Principles for Purposeful Business.” London. https://www.thebritishacademy.ac.uk/publications/future-of-the-corporation-principles-for-purposeful-business/.

[CR58] Mayer, C. (2013). *Firm commitment: Why the corporation is failing us and how to restore trust in it*. Oxford University Press.

[CR59] Mayer, C. (2018). *Prosperity. Better Business Makes the Greater Good*. Oxford: Oxford University Press.

[CR60] Mayer, C. (2020). Ownership, agency, and trusteeship: an assessment. *Oxford Review of Economic Policy,**36*(2), 223–240. 10.1093/oxrep/graa006

[CR61] Mayer, C. (2021). The future of the corporation and the economics of purpose. *Journal of Management Studies,**58*(3), 887–901. 10.1111/joms.12660

[CR62] Mehrotra, Vikas, and Randall Morck. 2017. “Governance and Stakeholders.” In *The Handbook of the Economics of Corporate Governance*, edited by Benjamin E. Hermalin and Michael S. Weisbach, 637–83. 10.1016/bs.hecg.2017.11.004.

[CR63] Miller, D. (2020). Reconceiving the democratic boundary problem. *Philosophy Compass,**15*(11), 1–9.

[CR64] Mitchell, R. K., Weaver, G. R., Agle, B. R., Bailey, A. D., & Carlson, J. (2016). Stakeholder agency and social welfare: Pluralism and decision making in the multi-objective corporation. *Academy of Management Review,**41*(2), 252. 10.5465/amr.2013.0486

[CR65] Moriarty, J. (2014). The connection between stakeholder theory and stakeholder democracy: An excavation and defense. *Business and Society*. 10.1177/0007650312439296

[CR66] Pitkin, H. (1967). *The concept of representation*. The University of California Press.

[CR67] Pollman, Elizabeth. 2021. “The History and Revival of the Corporate Purpose Clause.” *Texas Law Review* forthcomin.

[CR68] Porter, Michael, and Mark Kramer. 2011. “Creating Shared Value.” *Harvard Business Review* Jan-Feb: 1–17.

[CR26] Purpose Foundation. 2017. “Steward-Ownership. Rethinking Ownership in the 21st Century.” https://purpose-economy.org/content/uploads/purposebooklet_en.pdf.

[CR69] Raelin, J. D., & Bondy, K. (2013). Putting the good back in good corporate governance: The presence and problems of double-layered agency theory. *Corporate Governance: An International Review,**21*(5), 420–435. 10.1111/corg.12038

[CR70] Sanders, A. (2022). Binding capital to free purpose: Steward ownership in Germany. *European Company and Financial Law Review,**19*(4), 622–653.

[CR71] Sanders, A., & Thomsen, S. (Eds.). (2023). *Enterprise Foundation Law in a Comparative Perspective*. Intersentia.

[CR72] Sanders, K. (2018). ‘Beyond Human Ownership’? property, power and legal personality for nature in aotearoa New Zealand. *Journal of Environmental Law,**30*(2), 207–234. 10.1093/jel/eqx029

[CR73] Segrestin, B., Hatchuel, A., & Levillain, K. (2020). When the law distinguishes between the enterprise and the corporation: The case of the new French law on corporate purpose. *Journal of Business Ethics*. 10.1007/s10551-020-04439-y

[CR74] Singer, A. (2018). *The form of the firm: A normative political theory of the corporation*. Oxford University Press.

[CR75] Sjåfell, Beate. 2018. “Redefining Agency Theory to Internalize Environmental Product Externalities.” In *Preventing Environmental Damage from Products. An Analysis of the Policy and Regulatory Framework in Europe*, edited by Eléonore Maitre-Ekern, Carl Dalhammar, and Hans Christian Bugge, 101–24. Cambridge: Cambridge University Press.

[CR76] Smith, N. C., & Rönnegard, D. (2016). Shareholder primacy, corporate social responsibility, and the role of business schools. *Journal of Business Ethics*. 10.1007/s10551-014-2427-x

[CR77] Stehr, P. (2022). The boundary problem in workplace democracy: Who constitutes the corporate demos? *Political Theory*. 10.1177/00905917221131821

[CR78] Stoelhorst, J. W., & Vishwanathan, P. (2024). Beyond primacy: A stakeholder theory of corporate governance. *Academy of Management Review,**49*(1), 107–134. 10.5465/amr.2020.0268

[CR79] Stout, L. (2012). *The shareholder value myth*. Berrett-Koehler Publishers.

[CR80] Strine, L. (2017). Corporate power is corporate purpose 1: Evidence from my hometown. *Oxford Review of Economic Policy,**33*(2), 176–187.

[CR81] Tallarita, Roberto. 2023. “AI Is Testing the Limits of Corporate Governance.” *Harvard Business Review*.

[CR49] Strine, L. E., Jr., Kovvali, A., & Williams, O. O. (2021). Lifting labor’s voice: A principled path toward greater worker voice and power within american corporate governance. *Minn. L. Rev.,**106*, 1325.

[CR82] Thomsen, S., & Kavadis, N. (2022). Enterprise foundations: Law, taxation, governance, and performance. *Annals of Corporate Governance*. 10.1561/109.00000031

[CR83] Tierney, B. (1982). *Religion, Law, and the Growth of Constitutional Thought*. Cambridge University Press.

